# Fluid balance during PICU stay in children after cardiac surgery with cardiopulmonary bypass

**DOI:** 10.1007/s00431-026-07231-8

**Published:** 2026-07-10

**Authors:** Victorien A. C. Luppes, Zala Pamir, Nico A. Blom, Mark G. Hazekamp, Peter P. Roeleveld, Nina A. M. Houben, Jacobien B. Eising, Ariane Willems, Arend D. J. ten Harkel

**Affiliations:** 1 Division of Pediatric Cardiology, Department of Pediatrics, Willem-Alexander Children’s Hospital, Leiden University Medical Center, Leiden, The Netherlands; 2 Department of Cardiothoracic Surgery, Leiden University Medical Center, Leiden, The Netherlands; 3 Division of Pediatric Intensive Care, Department of Intensive Care, Leiden University Medical Center, Leiden, The Netherlands; 4 Division of Neonatology, Department of Pediatrics, Willem-Alexander Children’s Hospital, Leiden University Medical Center, Leiden, the Netherlands; 5 Division of Pediatric Intensive Care, Department of Pediatrics, Queen Fabiola University Children’s Hospital, Brussels, Belgium

**Keywords:** Heart Defects Congenital, Cardiac Surgical Procedures, Intensive Care Units Pediatric, Fluid Balance, Fluid Overload, Postoperative Complications

## Abstract

**Background:**

Pediatric patients undergoing cardiac surgery are at risk for developing positive cumulative fluid balance (CFB). Our goal was to study postoperative CFB in this population, its explanatory variables, and its effect on clinical outcomes during pediatric intensive care unit (PICU) stay and follow-up.

**Methods:**

Retrospective single center study of 200 consecutive children undergoing congenital heart surgery with cardiopulmonary bypass (CPB). A clinically relevant CFB was defined as ≥ 5% at the end of postoperative day 1 (CFB POD1 ≥ 5%).

**Results:**

A CFB POD1 ≥ 5% was observed in 36% of the patients. Lower weight and longer CPB time were identified as explanatory variables for developing CFB POD1 ≥ 5%. Patients with CFB POD1 ≥ 5% showed more acute kidney injury, higher vasoactive inotropic scores, and prolonged invasive ventilation and PICU length of stay (LOS). A 1% increase in CFB POD1 lengthened PICU LOS by 0.8 days (CI 0.588 – 1.001, *p* < 0.001). Furthermore, patients with CFB POD1 ≥ 5% had lower cardiac event free survival time (*p* < 0.001).

**Conclusions:**

This study confirms that even a minor positive CFB ≥ 5% is common in pediatric cardiac patients and associated with adverse PICU outcomes. Lower body weight and prolonged CPB duration increased the risk for positive CFB, warranting close fluid monitoring. Positive CFB was also associated with cardiac events during long-term follow-up and may help identify patients at increased risk for a complicated clinical trajectory, underscoring the importance of timely recognition and structured cardiac follow-up.

**Table:** 

**What is Known:** • *Positive cumulative fluid balance (CFB) is associated with adverse outcomes in critically ill pediatric patients, including those undergoing congenital heart surgery, though causality remains unclear.* • *Evidence on optimal fluid management and clinically relevant CFB thresholds remains limited.*
**What is New:** • *This study confirms that a seemingly minor CFB of 5% is common and associated with adverse outcomes in a heterogenous pediatric cardiac cohort.* • *Positive CFB was additionally associated with adverse long-term outcomes, identifying positive CFB as a potential early marker of a complicated clinical course.*

## Introduction

A positive cumulative fluid balance (CFB) occurs frequently in children admitted to the pediatric intensive care unit (PICU) after cardiac surgery [1–10]. It can be described as a state of hypervolemia, edema, and excessive weight gain [11]. A positive CFB is associated with worse outcomes, including prolonged mechanical ventilation and PICU length of stay (LOS), low cardiac output syndrome, and mortality [1–5]. Furthermore, a positive CFB is associated with acute kidney injury (AKI) and renal replacement therapy (RRT) [6–8]. The degree of positive CFB that is related to adverse events is often referred to as fluid overload (FO) [12].

Pediatric patients undergoing congenital heart surgery are at increased risk of developing a positive CFB due to several factors, including pre-existing cardiac impairment and cardiopulmonary bypass (CPB) during surgery. The CPB contributes to postoperative inflammation, capillary leakage, and decreased renal function [13]. This results in a predisposition to develop positive CFB before entering the PICU. Postoperative positive CFB is further aggravated when postoperative fluid resuscitation is necessary. In the absence of evidence, clinical practice and guidelines regarding optimal postoperative fluid management vary worldwide [14, 15]. In our pediatric cardiac population, we defined a threshold of ≥ 5% positive CFB as clinically relevant early after surgery according to existing literature [2, 6, 9, 16].

We previously studied CFB in univentricular pediatric patients undergoing Fontan completion and found that positive CFB was associated with both short- and long-term complications [17]. In the present study, we aimed to investigate postoperative CFB in a broader pediatric population undergoing any congenital heart surgery with CPB, thereby expanding the applicability of our findings to a wider patient group. The primary objective of this study was to determine the incidence of postoperative positive CFB ≥ 5% in this population. Secondary objectives were to identify explanatory variables associated with the development of positive CFB ≥ 5%, to evaluate the association between positive CFB ≥ 5% and outcomes during PICU stay, and to assess the association between positive CFB ≥ 5% and cardiac events during long-term follow-up.

## Methods

This retrospective cohort study was conducted at a tertiary care center in the Netherlands. All patients younger than 18 years who underwent congenital heart surgery with CPB and were admitted to the PICU from January 2019 until December 2020, were included. To avoid duplication, only the major corrective surgery was included if patients underwent more than 1 cardiac surgery during the study period. The protocol was approved by the Institutional Review Board (IRB) of the department of Intensive Care and Anesthesiology of the Leiden University Medical Center (N028). Patient informed consent was waived. Procedures were followed in accordance with the ethical standards of the responsible IRB and the 1975 Helsinki Declaration.

In addition to baseline demographics, data collection comprised various preoperative, intraoperative and postoperative variables. Preoperative data included hemoglobin and serum creatinine levels. The Risk Adjustment in Congenital Heart Surgery-1 (RACHS-1) methodology was used [18]. Collected intraoperative data included CPB time, aortic cross-clamp (AOX) time, minimum body temperature, amount of ultrafiltration (UF), and total administered erythrocytes (bolus only, no priming) and fresh frozen plasma (FFP). Postoperative data collected included duration of invasive ventilation, PICU LOS, and base excess measured on the day of surgery (DOS) at approximately 18:00 (± 3 h). Additionally, the highest lactate, lowest serum albumin, and highest serum creatinine during PICU stay were recorded. The vasoactive–inotropic score (VIS) was calculated during PICU stay using the following method: maximum dobutamine + (100 × maximum epinephrine) + (10 × maximum milrinone) + (100 × maximum norepinephrine) [19]. Requirement for postoperative ECMO support was also recorded. Acute kidney injury was defined using the Acute Kidney Injury Network (AKIN) definition [20, 21]. Preoperative serum creatinine levels were used as baseline and highest serum creatinine levels were considered during PICU stay. All postoperative PICU variables were analyzed during total PICU stay, from PICU admission after cardiac surgery until PICU discharge. Standard care included fluid restriction to 50–60% of maintenance requirements during the first 24 h after surgery. Fluid boluses were administered only when clinically indicated, and diuretics were routinely initiated from POD1 in hemodynamically stable patients. Prophylactic peritoneal dialysis was not part of standard care.

The CFB was calculated based on the daily fluid balance: [(total fluid in (L)–total fluid out (L))/preoperative weight (kg)] × 100% [22]. Fluids were cumulatively assessed for each day separately, starting at arrival at PICU on the DOS up to postoperative day (POD) 7. We defined a clinically relevant positive CFB as ≥ 5% at the end of POD1 (CFB POD1 ≥ 5%), which included the fluid balance on the DOS and POD1. This definition is based on the common occurrence of a high positive CFB shortly after surgery and is consistent with existing literature [2, 6, 9, 16].

The development of cardiac events was obtained from medical records from POD2 until January 2026. Cardiac events were analyzed by CFB POD1 category (< 5% vs ≥ 5%). Follow-up time for Kaplan–Meier analysis was set at POD2, ensuring that outcome assessment occurred after exposure classification and thereby preventing immortal time bias. Cardiac events were defined as a composite outcome of mortality, redo cardiac surgery, cardiac-related PICU re-hospitalization, and cardiac-related ward re-hospitalization. In patients presenting multiple cardiac events during follow-up, only the first event was considered in the analysis. We defined redo cardiac surgery as any major cardiac-related surgery or catheter-based intervention occurring on or after POD2. Minor cardiac surgeries such as placement of thoracic drains, drainage of pericardial effusion, delayed closure of the sternum, and diagnostic heart catheterizations were excluded. Cardiac-related PICU re-hospitalization was defined as any cardiac-related PICU re-hospitalization after discharge from the PICU. Cardiac-related ward re-hospitalization was defined as any clinically significant hospital admission to a general ward with a primary cardiac diagnosis requiring therapeutic intervention, occurring after discharge from the index hospitalization. Emergency department visits not resulting in hospitalization were excluded. Two pediatric cardiologists independently reviewed the cardiac events to classify minor and major surgeries as well as clinically relevant hospitalizations.

## Statistical analysis

Data analysis was performed using IBM SPSS Statistics version 29.0.0.0 and figures were created using GraphPad Prism version 10.6.1 and MATLAB version 2022b (MathWorks). Statistical significance was set at *p* < 0.05. Normally distributed data are presented as means (standard deviations), while non-normally distributed data are presented as medians [interquartile range]. Categorical data are presented as frequencies and percentages. Normality was assessed using the Shapiro–Wilk test. Data were compared between CFB cohorts using t-tests or Mann–Whitney U tests for continuous variables, and chi-square tests for categorical variables. Fisher’s exact test was used when expected cell counts were small.

Logistic regression was used to assess explanatory variables for CFB POD1 ≥ 5%. Variables associated with CFB POD1 ≥ 5% in univariate analysis (*p* < 0.1) were included in multivariable logistic regression analysis using backward selection. A post-hoc logistic regression analysis was performed using the variables retained in the final model to prevent unwanted missing cases due to covariates not selected in stepwise regression. Model fit was assessed using the Cox & Snell and Nagelkerke R^2^ statistics.

Linear regression was used to assess the association between CFB POD1 and PICU LOS. Variables associated with PICU LOS in univariate analyses (*p* < 0.1) were included in multivariable linear regression analysis using backward selection. Regression assumptions were assessed before analysis. A post-hoc multiple linear regression analysis was performed using the variables retained in the final model.

Kaplan–Meier log-rank pooled comparison was used to analyze cardiac events in patients with < 5% and ≥ 5% CFB POD1. For patients scheduled for staged repair procedures, follow-up was censored at the date of the planned redo surgery with CPB, provided that no event had occurred beforehand. Patients were excluded from follow-up if they died or underwent redo surgery with CPB before POD2, which prevented the assessment of long-term outcomes of the indexed surgery. Subsequently, a multivariable Cox proportional hazards regression was performed to account for baseline differences in RACHS-1 category, weight, and CPB duration.

## Results

**Fig. 1:**
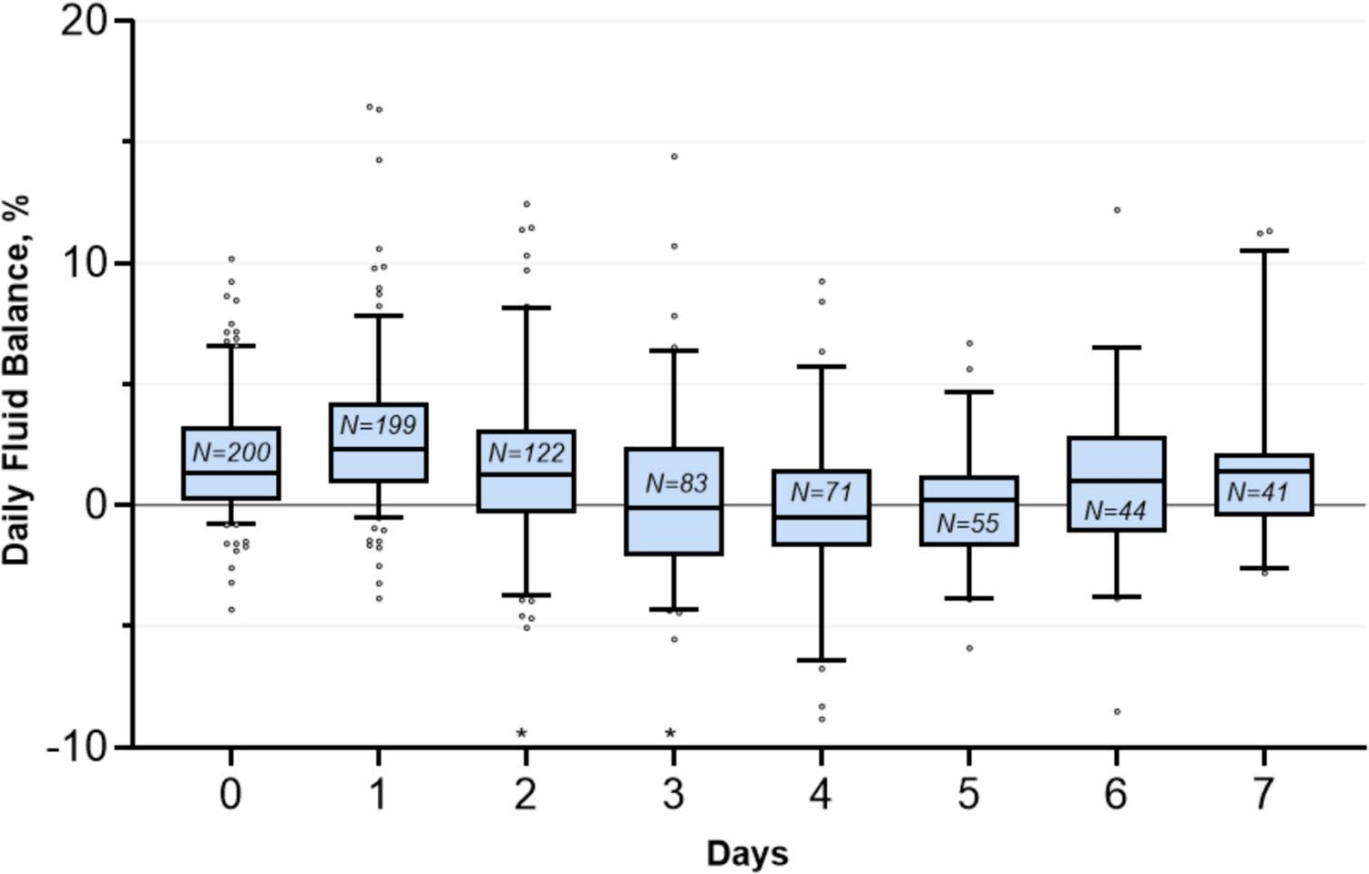
Daily fluid balance for each day separately, from the day of surgery up to postoperative day 7

This study included 200 consecutive patients who underwent congenital heart surgery with CPB and were subsequently admitted to the PICU. Median age was 15 [4–57] months and median weight was 10 [5–18] kg. Of the 200 patients, 113 (57%) were male, 22 (11%) were born premature, and 38 (19%) had a chromosomal abnormality. The RACHS-1 distribution was: category 1 (*n* = 20, 10%), category 2 (*n* = 74, 37%), category 3 (*n* = 88, 44%), category 4 (*n* = 15, 8%), and category 6 (*n* = 3, 2%). Preoperative mean hemoglobin levels were 8 (1) mmol/L and median serum creatinine levels were 27 [20–38] µmol/L. The daily fluid balance is shown in Fig. 1 for each day separately, starting from DOS up to POD7. The fluid balance tends to peak on POD1, whereas it stabilizes and returned to baseline thereafter.

### Demographics and clinical characteristics by CFB POD1

A majority of 128 patients had a CFB POD1 < 5% and 72 patients had a CFB POD1 ≥ 5%. Patients in the CFB POD1 ≥ 5% group were significantly younger and lower in weight (Table 1). Almost all patients with RACHS-1 score 1 had CFB POD1 < 5%. Other RACHS-scores did not differ significantly between groups. Patients with CFB POD1 ≥ 5% had significantly longer CPB and AOX durations and lower minimum intraoperative temperatures. They also had significantly higher volumes of administered erythrocytes, FFP, and performed UF during surgery.

**Table 1:** Demographics and clinical characteristics by CFB POD1

	CFB POD1 < 5% *n* = 128	CFB POD1 ≥ 5% *n* = 72	*p*-value
Demographics and preoperative			
Age, months	39 [6–96]	6 [3–16]	< 0.001
Male, n (%)	71 (56)	42 (58)	0.695
Weight, kg	14 [7–27]	7 [4–10]	< 0.001
Prematurity, n (%)	8 (6)	14 (19)	0.004
Chromosomal abnormality, n (%)	23 (18)	15 (21)	0.620
Hemoglobin, mmol/L	8 (1)	8 (1)	0.842
Creatinine, µmol/L	30 [22–42]	23 [18–31]	< 0.001
RACHS-1 category			0.027
Category 1, n (%)	19 (15)	1 (1)	0.001
Category 2, n (%)	44 (34)	30 (42)	0.305
Category 3, n (%)	56 (44)	32 (44)	0.924
Category 4, n (%)	7 (6)	8 (11)	0.146
Category 6, n (%)	2 (2)	1 (1)	1.000
Intraoperative			
CPB, min	89 [65–151]	124 [87–166]	0.002
AOX, min	50 [36–94]	80 [47–112]	0.002
Minimum temperature, °C	30 [28–33]	28 [28–32]	< 0.001
Administered erythrocytes, mL/kg	3 [0–10]	10 [3–17]	< 0.001
Administered FFP, mL/kg	0 [0–8]	8 [3–13]	< 0.001
UF, mL/kg	39 [8–73]	69 [30–99]	< 0.001
Postoperative			
CFB POD1, %	2 [1–4]	8 [6–11]	< 0.001

*CFB POD1* cummulative fluid balance at the end of postoperative day 1, *n* number of patients, *RACHS-1* risk adjustment for congenital heart surgery, *CPB* cardiopulmonary bypass, *AOX* aortic cross-clamp, *FFP* fresh frozen plasma, *UF* ultrafiltration

### Explanatory variables for CFB POD1 ≥ 5%

Univariate analysis demonstrated that younger age, lower weight, prematurity, higher RACHS-1 score, prolonged CPB time, higher total administered erythrocytes and FFP during surgery, and more UF during surgery were associated with an increased risk of developing CFB POD1 ≥ 5% (Table 2). Age was not further analyzed due to its multicollinearity with weight. Only weight and CPB time remained significant explanatory variables for developing CFB POD1 ≥ 5% in the multivariate logistic regression model. The model explained 23% to 32% of the variance in CFB POD1 depending on the method used (Cox & Snell R^2^ versus Nagelkerke R^2^). The classification accuracy was 73%.

**Table 2:** Logistic regression for independent variables associated with CFB POD1 ≥ 5%

	OR	95% CI	*p*-value
Univariate analysis			
Age, months	0.978	0.968–0.988	< 0.001
Weight, kg	0.912	0.873–0.952	< 0.001
Prematurity	3.621	1.438–9.117	0.006
RACHS-1 category	1.377	0.985–1.925	0.061
CPB, min	1.006	1.001–1.010	0.018
Administered erythrocytes, mL/kg	1.063	1.027–1.100	< 0.001
Administered FFP, mL/kg	1.058	1.019–1.098	0.003
UF, mL/kg	1.009	1.003–1.015	0.002
Multivariate analysis			
Weight, kg	0.905	0.866–0.946	< 0.001
CPB, min	1.010	1.004–1.017	< 0.001
Prematurity	2.713	0.973–7.567	0.057

*CFB POD1* cummulative fluid balance at the end of postoperative day 1, *OR* odds ratio, *CI* confidence interval, *RACHS-1 risk adjustment for congenital heart surgery, CPB* cardiopulmonary bypass, *FFP* fresh frozen plasma, *UF* ultrafiltration

### Effect of CFB POD1 ≥ 5% on clinical outcomes during PICU stay

As set out in Table 3, patients with CFB POD1 ≥ 5% required significantly longer invasive ventilation and had longer PICU LOS. They also had significantly higher VIS scores and lower lowest serum albumin levels. Postoperative ECMO support did not differ between groups. According to the AKIN criteria, 50 patients (25%) in the overall cohort were diagnosed with AKI. Patients with ≥ 5% CFB POD1 had significantly more AKI, although only two patients required RRT. The in-hospital mortality rate was 1.5%, with all cases occurring in the CFB POD1 ≥ 5% group. One additional patient died six days after hospital discharge and was therefore not included in the in-hospital mortality rate.

**Table 3:** Clinical PICU outcomes in patients with CFB POD1 < 5% and ≥ 5%

PICU outcome	CFB POD1 < 5% *n* = 128	CFB POD1 ≥ 5% *n* = 72	*p*-value
PICU LOS, days	1 [1–3]	5 [2–12]	< 0.001
Invasive ventilation, hours	5 [3–12]	34 [6–162]	< 0.001
Maximum lactate, mmol/L	1.6 [1.4–2.2]	2.0 [1.7–2.9]	< 0.001
Lowest serum albumin g/L	37 [33–40]	28 [24–34]	< 0.001
Base excess, mmol/L *	−6 [−7,−4]	−6 [−8,−4]	0.125
Highest creatinine, µmol/L	35 [27–48]	37 [28–53]	0.442
Renal replacement therapy, n (%)	0 (0)	2 (3)	0.128
AKIN stage, n = 196			
No AKI, n (%)	115 (90)	33 (47)	< 0.001
Stage 1, n (%)	11 (9)	14 (20)	0.021
Stage 2, n (%)	2 (2)	19 (27)	< 0.001
Stage 3, n (%)	0 (0)	4 (6)	0.015
VIS score	5 [0–12]	18 [8–35]	< 0.001
ECMO support, n (%)	1 (1)	4 (6)	0.057
In-hospital mortality, n (%)	0 (0)	3 (4)	0.045

*CFB POD1* cummulative fluid balance at the end of postoperative day 1, *n* number of patients, *PICU* pediatric intensive care unit, *PICU LOS* pediatric intensive care unit length of stay, *AKIN* Acute Kidney Injury Network, *AKI* acute kidney injury, *VIS* vasoactive–inotropic score, *ECMO* extracorporeal membrane oxygenation. *Base excess measured on the day of surgery at approximately 18:00 (± 3 h)

### Risk factors for prolonged PICU LOS

Univariate linear regression analysis outlined potential predictors for prolonged PICU LOS which are summarized in Table 4. In the multivariate analysis, the RACHS-1 score remained a significant covariate for PICU LOS, in addition to CFB POD1. In the final model, the administration of erythrocytes during surgery was the only non-significant variable. Adjusted R^2^ for the final model was 0.336. When evaluating standardized coefficients, CFB POD1 makes the strongest unique contribution to PICU LOS. The standardized coefficient of CFB POD1 was 0.469, compared to 0.199 for the RACHS-1 score. A 1% increase in CFB POD1 lengthened the PICU LOS by 0.8 days (CI 0.588–1.001, *p* < 0.001).

**Table 4:** Linear regression on the effect of CFB POD1 on PICU LOS, adjusted for confounding variables

	Unstandardized β	95% CI	*p*-value
Univariate analysis			
CFB POD1, %	0.912	0.712–1.112	< 0.001
Age, months	−0.029	−0.047—−0.011	0.002
Prematurity	2.891	−0.315 – 6.097	0.077
Weight, kg	−0.092	−0.151—−0.032	0.003
RACHS-1 category	2.388	1.291–3.485	< 0.001
CPB, min	0.034	0.018–0.049	< 0.001
Administered erythrocytes, mL/kg	0.238	0.136–0.341	< 0.001
Administered FFP, mL/kg	0.192	0.082–0.301	< 0.001
UF, mL/kg	0.023	0.003–0.042	0.023
Multivariate analysis			
CFB POD1, %	0.795	0.588–1.001	< 0.001
RACHS-1 category	1.627	0.674–2.580	< 0.001
Administered erythrocytes, mL/kg	0.087	−0.007 – 0.182	0.070

*CI confidence interval, CFB POD1* cummulative fluid balance at the end of postoperative day 1, *RACHS-1* risk adjustment for congenital heart surgery, *CPB* cardiopulmonary bypass, *FFP* fresh frozen plasma, *UF* ultrafiltration

### Long term follow-up of cardiac events

**Fig. 2:**
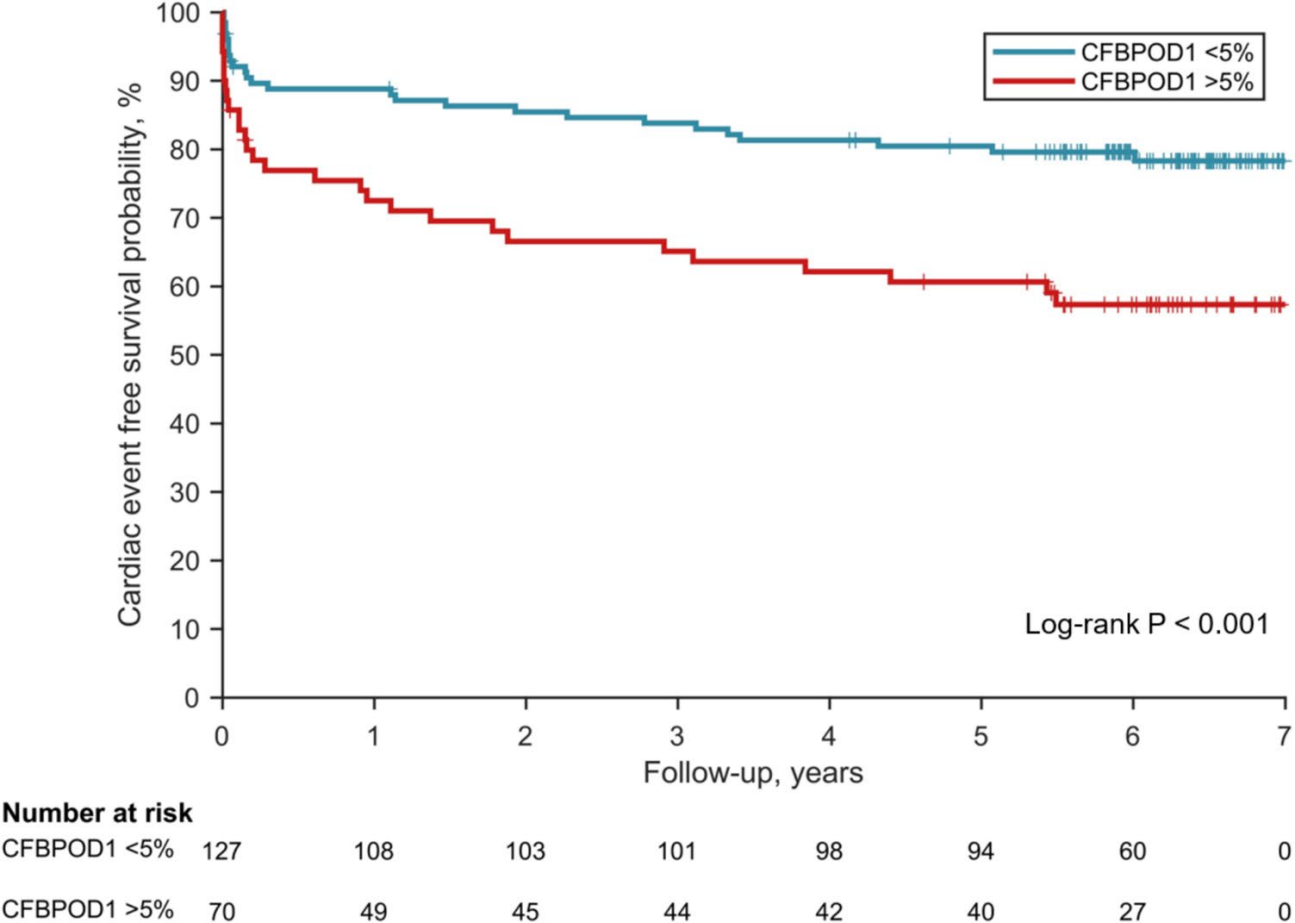
Kaplan–Meier curves for cardiac event free survival in patients with CFB POD1 < 5% and ≥ 5%

Cardiac event free survival time for 197 patients is presented in Fig. 2. Three patients were excluded from follow-up due to events that had occurred before POD2. These events included ECMO (*n* = 3) followed by death in 1 patient. Overall mean survival time was 5.3 years (95% CI 5.0–5.7). Patients with ≥ 5% CFB POD1 had a significantly shorter event-free survival time (*p* < 0.001). A total of 55 cardiac events occurred, of which 1 death, 41 redo cardiac surgeries, 4 PICU re-hospitalizations, and 9 cardiac-related ward hospitalizations. The remaining 142 patients were censored due to loss to follow-up (*n* = 8), planned redo surgery with CPB (*n* = 4), or end of follow-up (*n* = 130). In multivariable Cox regression analysis, CFB ≥ 5% remained associated with an increased risk of cardiac events (HR 2.0, 95% CI 1.1–3.7, p = 0.028), after adjustment for RACHS-1 category, weight, and CPB duration.

## Discussion

In this heterogenous cohort of children undergoing congenital heart surgery with CPB, 36% had a CFB POD1 ≥ 5%. Lower weight and longer CPB time were identified as explanatory variables. A CFB POD1 ≥ 5% was associated with prolonged invasive ventilation and PICU LOS, as well as a higher incidence of AKI. Patients with CFB POD1 ≥ 5% had a shorter cardiac event-free survival time during long-term follow-up. The association of a positive CFB with adverse long-term outcomes may support its role as an early marker of disease severity and a complicated clinical trajectory.

There is a great variability in reported incidences of clinically relevant positive CFB (10–65%) in children and neonates undergoing cardiac surgery [8, 10]. The incidence of positive CFB in the present study is lower compared to the study by Selewski et al. who studied a large cohort of critically ill children admitted to the PICU [16]. A higher incidence of positive CFB in cardiac patients could be expected due to poor preoperative status, the CPB-related inflammatory response, and fluid resuscitation during PICU stay to optimize preload and maintain adequate cardiac output [13, 23]. However, our reported incidence is in line with previous studies, in which approximately 30% of children with CHD developed early postoperative CFB ≥ 5% [6, 9].

In addition to differences in incidence, reported CFB cutoff values also vary considerably across studies, ranging from 2.7% to 20% [5]. We used a cutoff value of 5% on POD1 in line with previous literature [2, 6, 9, 16]. In our cohort, CFB POD1 ≥ 5% was associated with adverse outcomes, including AKI, prolonged postoperative invasive ventilation, and longer PICU LOS. Furthermore, regression analysis identified CFB POD1 as a strong predictor of PICU LOS. Two recent systematic reviews and meta-analyses in critically ill children concluded that positive CFB is common and associated with adverse outcomes including mortality [24, 25]. Similar associations have also been reported in children undergoing congenital heart surgery [3, 5–7, 9, 10, 17]. Extending these findings, we demonstrate that patients with CFB POD1 ≥ 5% experienced shorter cardiac-event free survival time during long-term follow-up. This relationship with long-term cardiac events may be explained by CFB acting as an early clinical marker of a complicated trajectory, rather than as a direct causal factor for cardiac events occurring years later. The associations of positive CFB with younger age, lower weight, and prolonged CPB duration, as confirmed in our study, further support its role as a marker of disease severity and surgical complexity associated with a complicated clinical course. Furthermore, residual confounding cannot be excluded. Although causality cannot be established or ruled out, patients with positive CFB should be recognized as a high-risk group requiring appropriate long-term follow-up.

Manipulating fluid balance alone may not improve long-term outcomes in this patient group, as positive CFB may reflect a complicated clinical trajectory rather than a direct causal factor. Nevertheless, previous studies have reported promising results with restrictive fluid management strategies [26–29]. In addition, fluid removal strategies such as diuretics and prophylactic peritoneal dialysis have shown efficacy in children undergoing cardiac surgery with CPB [30–33]. While early aggressive fluid resuscitation can be crucial in selected patient populations [34], critically ill children often receive substantial fluid volumes as part of standard care, including nutrition, intravenous analgesics and sedatives, and maintenance fluids. This may account for up to 30% of total fluid intake, compared with less than 10% attributable to resuscitation fluids [35]. Although of utmost importance, evidence regarding restrictive fluid management and active fluid removal strategies remains limited [36]. Positive CFB likely reflects overall illness severity, and it remains unknown whether a more conservative fluid management strategy can improve outcomes. However, a complicated PICU course is itself associated with adverse long-term outcomes [37]. Consequently, optimization of fluid balance may reduce PICU-related complications, such as prolonged invasive ventilation and PICU length of stay, and thereby still improve long-term outcomes. Large randomized controlled trials are therefore needed to determine the role of fluid management strategies in critically ill children, including those with congenital heart disease.

### Limitations and strengths

A first limitation is the retrospective and single-center character of the study, which limits causal inference and reduces external validity. Residual confounding by unmeasured factors such as preoperative volume status and nutritional status cannot be excluded. While the heterogeneous study population improves the generalizability of our findings, it may also have increased the potential for residual confounding. Second, we calculated CFB based on the fluid balance methodology and did not incorporate insensible fluid losses. This is in contrast to CFB calculation based on daily weight that does incorporate insensible losses [38]. Differences between fluid-based and weight-based methods may yield different estimates of CFB and may result in different patient classifications around predefined thresholds, as demonstrated in unpublished data [39]. However, accurate weight assessment is challenging in the PICU setting. Third, we used serum creatinine levels to calculate AKIN stages and identify AKI. Serum creatinine levels could be underestimated in cases of positive CFB due to dilution of the serum creatinine. This may have underestimated the prevalence of AKI in our study [40–42]. Strength of our study is that we thoroughly investigated CFB in a representative cohort of 200 cardiac patients with various ages and multiple cardiac anomalies. By linking positive CFB during PICU admission to cardiac events in long-term follow-up, our study provides novel insights.

## Conclusions

Over one-third of children undergoing congenital heart surgery with CPB developed a positive CFB ≥ 5% early postoperatively. Positive CFB was associated with adverse PICU outcomes. It was also associated with cardiac events during long-term follow-up and may contribute to early risk stratification of patients at risk for a complicated clinical course. Patients with positive CFB should therefore be recognized as a high-risk group requiring appropriate long-term follow-up. Nonetheless, further prospective and interventional research is essential to establish causality and determine whether optimized fluid management improves outcomes in this vulnerable population.

## Data Availability

The data presented in this study are available upon reasonable request from the corresponding author.

## Abbreviations

AKI: Acute Kidney Injury
AKIN: Acute Kidney Injury Network
AOX: Aortic Cross-Clamp
CFB: Cumulative Fluid Balance
CPB: Cardiopulmonary Bypass
DOS: Day of Surgery
ECMO: Extracorporeal Membrane Oxygenation
FFP: Fresh Frozen Plasma
FO: Fluid Overload
IRB: Institutional Review Board
LOS: Length of Stay
PICU: Pediatric Intensive Care Unit
POD: Postoperative Day
RACHS-1: Risk Adjustment in Congenital Heart Surgery-1
RRT: Renal Replacement Therapy
UF: Ultrafiltration
VIS: Vasoactive–Inotropic Score
